# The Management of Strabismus Convergens*Clinical remarks before the class of the New York Post Graduate Medical School.

**Published:** 1883-06

**Authors:** B. St. John Roosa


### The Management of Strabismus Convergens.*

By B. St. John Roosa, M. D., LL. D.

The management of strabismus convergens was formerly supposed to be a very simple affair. When Donders showed its relations to a short anterio-posterior diameter of the eyeball, a new importance was attached to it. It was found that mere division of the *rectus internus* was not always sufficient to cure the squint. The ciliary muscles and the internal recti act together. If you paralyze the ciliary muscle by the use of atropia, you at the same time lessen the power of the internal rectus. In the use of this drug, therefore, a means of dispensing with the operation was found by some authorities. But full trial has proved that these hopes of cure without cutting are usually illusory, and the division of the muscle has as firm a hold upon the profession as ever. But the use of atropia *after* the operation, the use of glasses *after* the operation are often—yes, generally necessary, to effect a cure.

In the forty cases that have been operated upon in this division of the hospital since November 1st, atropia and glasses have been required in nearly every case. Any one may soon acquire the mechanical skill to take up the internal rectus on a hook and to divide it close to its insertion with the scissors; but to learn what is known about strabismus convergens, and to appreciate the gaps in our knowledge, will require some months of careful study and close observation. It is not the simple subject that Diffenbach thought it. Perhaps I may say, it is not yet as clearly understood as even Donders supposed his observations had made it. If hypermetropia be the chief cause of convergent squint, why do so small a proportion of hypermetropics squint? A large percentage of the people of the world who are not *myopic* are *hypermetropic*. Yet very few of the latter squint. Why is one operation sufficient to cure some cases of marked strabismus, while two or three, or even four, are sometimes

* Clinical remarks before the class of the New York Post Graduate Medical School.

needed for some, in which the deformity is slight? I am not able to tell beforehand in which cases I shall be obliged to operate twice or three times. I always tell the friends of the patient beforehand, that to get a good result they must leave the case to my observation for at least six weeks.

You have observed that although I make a free incision into the conjunctiva and into Tenor's capsule, that I never use a suture after the operation. I do not *usually* find any difficulty afterward in the way of a cicatrix or granulation. It is easier to snip off a granulation if it came, than to take out a suture. The child generally regards the cutting a suture as another operation, and dreads it accordingly. We have had but one case of inflammation of the edges of the wound this winter. In all the cases, except those just performed, I finally "straightened the eyes." In a few months after the operation, I usually advise that the use of glasses, as for young hypermetropics in general, be given up, except for close work. Their use is, however, essential in the first few weeks after the operation is performed. I know of no subject in ophthalmology that will better repay study, and in which there is a better prospect of new contributions to our knowledge, than this of the management of strabismus convergens.—*The Planet.*

